# Diseases of Children

**Published:** 1905-04-22

**Authors:** 


### DISEASES OF CHILDREN.

**Syphilitic Synovitis.** — Dr. Melville Dunlop[1] directs attention to this form of arthritis, which is not particularly rare in childhood, but which is probably often erroneously diagnosed as tuberculous or rheumatic. It is usually the result of congenital syphilis, and occurs as a rule between the ages of eight and fifteen years. The leading feature is a painless effusion into both knee-joints. Sometimes one joint is affected before the other, and sometimes several large joints are affected, but the frequency with which the knee-joints alone are the seat of disease is a striking and unexplained characteristic. There is no acute inflammation, no tendency to involvement of the cartilage or bone, and no disturbance of function. Other symptoms of congenital syphilis can usually be determined, and the frequent association of this form of arthritis with interstitial keratitis has been noted by several observers. The similarities between these affections are very striking, as both are chronic in their course, produce little or no destructive changes in the tissues involved, and do not respond to mercurial treatment. In some cases involvement of the cartilages or of the bone itself have been described, but these are rare. Surgical measures are not necessary and do not expedite the recovery. Many cases will recover under a course of mercury and iodide of potassium, but others, as already stated, pursue a very chronic course irrespective of treatment, although these also usually terminate in complete recovery.

**Convulsions.**—The view that convulsions occurring during the first few days of life are commonly due to intra-cranial hæmorrhage is combated by Dr. Henry Ashby.[2] He does not deny this possibility, but holds that a much more frequent cause is gastro-intestinal disturbance, due to artificial feeding, and that in some cases epilepsy, the result of fœtal brain disease, is the chief factor. The fits may recur from hour to hour, and the exhaustion may be extreme. They may be one-sided and yet reflex in origin. Probably the starting-point is an inflamed and over-active colon; possibly the absorption of toxins may have some influence on the nerve centres. Infection by the mouth is a much commoner source of trouble than when the infant is breast fed. At a later stage dentition is by many held responsible for fits, epilepsy, and idiocy; but here again Dr. Ashby holds that the commonest cause is gastro-intestinal disturbance, accompanied by colic and griping, due in many cases at least to overfeeding with cows' milk. In other cases bronchial or naso-pharyngeal catarrh, or middle-ear suppuration, may be the exciting cause. One type of convulsion occurring during infancy is distinctly of the Jacksonian type, and is due to a syphilitic softening of the brain. An infant a few months old, who has suffered from coryza, erythema, and epiphysitis, begins to suffer from slight, one-sided convulsions. There is a momentary loss of consciousness or only a dazed look. Later the twitchings may affect the opposite side, and then gradually a contraction of the flexures of the arm and leg first affected takes place, and the infant shows signs of less and less intelligence. Patchy degeneration of the grey matter of the cortex, with degeneration of the arteries, and later cerebral sclerosis are the underlying pathological changes.

**Infant Feeding.**—It has been pointed out by Dr. Wright and others that if the lime salts in cows' milk are partly precipitated, the curd which is subsequently formed by the addition of rennet is delayed in time and less dense than that formed in milk not so treated. Wright suggested the use of citrate of soda for this purpose. Dr. F. J. Poynton [3] has been using citrate of soda in this manner for some time, and believes that it is useful. He gives one grain of citrate of soda for each ounce of milk in the meal. The taste of the milk is not perceptibly altered, there is no alkaline change in the milk, and no fear need be entertained of the development of rickets or impaired bony tissues from the partial precipitation of the lime salts. The only drawback he has noticed has been the occurrence of constipation. Dr. G. F. Still [4] considers that a large majority of the failures in infant feeding are due to insufficient dilution of milk. He suggests the following as a good three-ounce mixture:—

| *Mixture.* | *Resulting Percentages.* |
|---|---|
| Milk, 6 drachms ... ... ... | Casein, 0·81 %. |
| Whey, 18 drachms ... ... ... | Lactalbumin, 0·75 %. |
| Cream (48 % fat), 1 drachm ... ... | Fat, 3·87 %. |
| Milk sugar, ½ level teaspoonful ... | Sugar, 6·50 %. |

The milk here supplies the casein, which is usually the part in excess in ordinary milk mixtures, and causes the difficulty in digestion. The whey supplies the lactalbumin, and is the natural diluent of milk. It is rather difficult to prepare for this purpose, for after having been separated from the milk it must be heated to a certain temperature to destroy the milk-curdling ferment, but not such a temperature as to coagulate the lactalbumin. The temperature employed should be between 150° and 155° F. The cream he refers to is centrifugal cream, which has an average strength of 48 per cent. of fat. The above mixture has much to recommend it when sufficient time and care can be devoted to its preparation. Dr. H. Dwight Chapin [5] has written a destructive criticism of the chemical methods of artificial feeding, and suggests that the biological element is more deserving of study. In breast-feeding there is a progressive development of the functions of the stomach, which is accompanied by a corresponding change in the natural supply. Sterilising, pasteurising, or heating cows' milk alters it chemically so that rennet will not act upon it readily. It is easy to make up a food that will contain the same quantities of fat, proteids, carbohydrates, etc., as maternal milk, but experience and study only will enable the infant-feeder to do what nature does automatically—adapt the food to the conditions actually present and ensure proper functional development. The account given by Dr. Aitchison Robertson [6] of the Edinburgh dairies is such as to make one wonder how any bottle-fed infants in that city survive. He purchased a large number of samples of milk at different shops, and in more than half of these it had reached a stage of putrefaction such as to preclude its use as a food for infants and young children. The excess of lactic acid produces gastric irritation, flatulence, and diarrhœa, and the vitality of the intestinal mucosa is so lowered as to allow of the penetration of tubercle bacilli, also introduced in the food. The presence of tubercle bacilli in the milk is easily explained by the tuberculosis-inducing conditions under which the cows live. In the city cowsheds there are 3,072 cows, which are never allowed out from the time they are bought as milkers. Practically each cow is kept there as long as she is giving a quantity of milk which pays, and this period may last for some years. The cowsheds are grossly overcrowded, overheated, badly ventilated, and destitute of sunshine. In fact the city authorities ought to be ashamed that such a state of affairs has been tolerated so long. Dr. Robertson remarks that medical men ought to be acquainted with these matters, and he gives a very full summary of the necessary reforms for the protection of infant life.

**The Treatment of Pneumonia.**—Dr. W. P. Northrup [7] holds very strong views on the subject of treating pneumonia by means of cold fresh air. He describes a case of broncho-pneumonia in an infant thus treated during an illness of 12 days, with complete recovery. The drugs used were castor-oil (one dose), four strychnine tablets, one-sixtieth grain each, and one drachm of whisky. The two windows and two doors of the bedroom were kept open day and night, and as the month was December the temperature in the room sometimes fell as low as 28° F. The nurses wore automobile fur coats, and the doctor seldom removed his overcoat at his visits. The child was of course kept warm by blankets, hot bottles, etc., and any cooling of the extremities was at once checked by hot foot-baths. The author may push his view regarding cold air too far, but, on the other hand, there has been too much of what may be termed the opposite method of treatment. This method is summarised by Dr. Northrup as follows: "*How to kill a baby with pneumonia.*—Crib in far corner of room with canopy over it. Steam-kettle, gas-stove (leaky tubing), room at 80° F. Many gas jets burning. Friends in the room, also the pug dog. Chest tightly enveloped in waistcoat poultice. If child's temperature is 105° make a poultice, thick, hot, and tight. Blanket the windows, shut the doors. If these do not do it, give coal-tar antipyretics and wait." There is far too much truth in this picture even in the present day. At a discussion [8] on pneumonia in New York a variety of opinions was expressed. Dr. J. E. Winters recommended that the child should be kept in a warm bed, in a room having a temperature of at least 72° F., and that no cold applications to the surface of the body should be used. Dr. R. G. Freeman laid stress on the diminution of the respiratory murmur as a valuable early sign in pneumonia, and had also noted that the disease might run its course without the presence of any distinct physical signs in the lungs.

[1] Edin. Med. Jour., Dec. 1904. [2] Lancet, Jan. 21, 1905. [3] Ibid., 1904, Vol. ii., p. 433. [4] Clin. Jour., Nov. 25, 1904. [5] Med. Rec., Dec. 12, 1903. [6] Scot. Med. and Surg. Jour., Feb. 1905. [7] Med. Rec., Feb. 18, 1905. [8] Ibid., Dec. 19, 1903.

(*To be continued.*)

